# The Overview of Perspectives of Clinical Application of Liquid Biopsy in Non-Small-Cell Lung Cancer

**DOI:** 10.3390/life12101640

**Published:** 2022-10-19

**Authors:** Aleksandra Bożyk, Marcin Nicoś

**Affiliations:** Department of Pneumonology, Oncology and Allergology, Medical University of Lublin, 20-059 Lublin, Poland

**Keywords:** NSCLC, liquid biopsy, circulating-free DNA, circulating-tumor DNA, personalized treatment

## Abstract

The standard diagnostics procedure for non-small-cell lung cancer (NSCLC) requires a pathological evaluation of tissue samples obtained by surgery or biopsy, which are considered invasive sampling procedures. Due to this fact, re-sampling of the primary tumor at the moment of progression is limited and depends on the patient’s condition, even if it could reveal a mechanism of resistance to applied therapy. Recently, many studies have indicated that liquid biopsy could be provided for the noninvasive management of NSCLC patients who receive molecularly targeted therapies or immunotherapy. The liquid biopsy of neoplastic patients harbors small fragments of circulating-free DNA (cfDNA) and cell-free RNA (cfRNA) secreted to the circulation from normal cells, as well as a subset of tumor-derived circulating tumor cells (CTCs) or circulating tumor DNA (ctDNA). In NSCLC patients, a longitudinal assessment of genetic alterations in “druggable” genes in liquid biopsy might improve the follow-up of treatment efficacy and allow for the detection of an early progression before it is detectable in computed tomography or a clinical image. However, a liquid biopsy may be used to determine a variety of relevant molecular or genetic information for understanding tumor biology and its evolutionary trajectories. Thus, liquid biopsy is currently associated with greater hope for common diagnostic and clinical applications. In this review, we would like to highlight diagnostic challenges in the application of liquid biopsy into the clinical routine and indicate its implications on the metastatic spread of NSCLC or monitoring of personalized treatment regimens.

## 1. Introduction

Lung cancer is the leading oncological issue and affects 2.1 million people worldwide annually. Despite personalized treatment regimens, such as molecularly targeted therapy and immunotherapy, the mortality of lung cancer remains very high and accounts for around 1.8 million deaths yearly [1]. For a standard diagnostics procedure of lung cancer, tissue sampling is required by the application of surgical procedures or bronchoscopy, which are considered to be invasive techniques. Samples obtained in these ways are sufficient for diagnostic purposes; however, their utility is limited in the monitoring of response to the treatment and for tracking disease progression [2]. It is mainly caused by the fact that re-sampling of the primary tumor at the moment of progression, in many cases, is impossible due to the advanced stage of the disease or poor clinical condition of the patients [3]. However, re-biopsy is a growing trend in oncology, especially after disease progression in patients with advanced or metastatic non-small-cell lung cancer (NSCLC), and it may shed light on the mechanism of resistance to therapy or improve the selection of NSCLC patients who may benefit from next-generation personalized regiments [4,5]. Particularly, the monitoring of genetic alterations in “druggable” genes such as *EGFR*, *ALK*, *ROS1*, or *PD-L1* might improve the follow-up of treatment efficacy, as well as allow us to detect an early progression before it is detectable in computed tomography [6]. Therefore, there is a pressing need to provide noninvasive procedures, and there are many premises for which peripheral blood, sampled as a liquid biopsy, could increase the healthcare in these deadly conditions [7].

The clinical application of liquid biopsy was already determined in many solid cancers for the early screening and monitoring of minimal residual disease (MRD) or acquired resistance to treatment [8]. There are cancers characterized by a low-circulating tumor DNA (ctDNA) concentration, such as in renal cell, brain, or prostate cancers, where the application of liquid biopsy is limited [9]; meanwhile, colorectal cancer (CRC), breast cancer (BC), melanoma indicate promising results for the implication of liquid biopsy into clinical routine [10].

In BC the high levels of ctDNA or plasmatic *HER2* gene amplification have been associated, respectively, with more aggressive course and anti-HER2-resistant disease [8,11]. Subsequently, both plasmatic hormone status and *HER2* amplification might predict the primary resistance to trastuzumab emtansine in metastatic BC patients [12]. Moreover, *PIK3CA* gene mutations in plasma may be a negative predictive biomarker for CDK4/6 inhibitors [13], while *ESR1* gene mutations have been related to aromatase resistance [8,14]. Recently, epigenetic alterations, such as DNA methylation, in the promoter regions of *SOX17*, *BRMS1*, and *CST6* genes showed an association with enhanced tumor metastasis and poor prognosis in BC patients [15,16,17], while methylation of *ESR1* correlates with treatment resistance to chemotherapeutic regimens such as everolimus and exemestane [17,18].

In CRC patients, liquid biopsy has especially demonstrated promise in the monitoring of MRD [8] or therapy response [17]. The clinical value of ctDNA for early CRC screening is limited [19]; on the other hand, the recurrence rate is higher if ctDNA is prospectively detectable before the treatment [10]. The biggest promise for liquid biopsy in CRC patients is related to the assessment of *KRAS* or *BRAF* mutations conferring resistance to treatment with anti-EGFR monoclonal antibodies [8,20]. In particular, it was revealed that *KRAS* subclones emerged in liquid biopsy 10 months before radiographic progression [21]. At the epigenetic level, some years ago, methylation of the *SEPT9* gene promoter was indicated to be a promising biomarker for early diagnosis of CRC [22]; however, this test did not distinguish optimally CRC from polyps or adenomas, and thus it has not replaced colonoscopy in early screening [23].

In melanoma, a liquid biopsy might represent a valuable tool for the detection of typical mutations in *BRAF* and *NRAS* genes, microsatellite alterations, and epigenetic modifications such as DNA methylation [8,24]. Several studies correlated a plasmatic high level of *BRAF* V600E substitution with shorter clinical outcomes [25,26]. Moreover, the plasmatic fractions of *BRAF* mutations measured in the first week following the initiation of immunotherapy [10] might predict the response to PD-1 or CTLA-4 inhibitors in advanced melanomas [8,27,28]. In the end, in ovarian-cancer pretreatment, ctDNA levels were more informative for cancer progression and its therapeutic response rather than the CA125 marker [10]. In pancreatic cancer, the plasmatic genetic landscape of copy-number alterations might have a predictive value for cancer progression [29], and the methylation of *VEGF* and *SFRP2* genes could impact angiogenesis [17].

Considering the wide spectrum of liquid biopsy impactions in many solid tumors, in this review, we discuss the main components of liquid biopsy and diagnostic challenges in the application of liquid biopsy into the NSCLC clinical routine. Furthermore, we indicate the implications of tumor-derived elements of liquid biopsy on a metastatic spread of NSCLC. Finally, we highlight the applications of liquid biopsy in the monitoring of personalized treatment of NSCLC that are already available in clinics or stays under consideration in clinical trials.

## 2. Liquid Biopsy—A Bit of Systematics

The history of liquid biopsy dates back to the 19th century, when Thomas Ashworth observed circulating tumor cells (CTCs) in a blood sample from an advanced oncologic patient with many distant metastases. Furthermore, circulating free nucleic acids were described in 1948 in healthy patients [30], whereas circulating DNA was detected for the first time in oncologic patients in 1977 [31]. The following years brought a discovery of liquid biopsy elements that can be assessed as diagnostic material [32]. The liquid biopsy of healthy cohorts carries the small fragments of circulating-free DNA (cfDNA) secreted to the circulation from normal cells [33]. However, in cancer patients, the liquid biopsy is represented by a subset of CTCs, circulating tumor DNA (ctDNA), and cell-free RNA (cfRNA) [34]. Each of these biomarkers can be used to determine a variety of relevant molecular or genetic information that demonstrates liquid biopsy as a comprehensive tool for understanding the biology of the tumor, monitoring the response to treatment, and studying intratumor heterogeneity [35].

**Figure 1:**
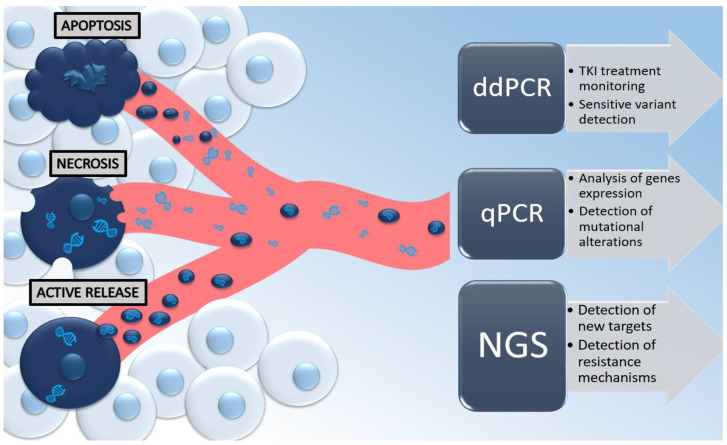
Mechanisms of tumor-derived DNA released in the bloodstream from the neoplastic cell and its clinical utility with the application of the most common genetic methods, such as ddPCR (digital-droplet PCR), qPCR (quantitative PCR), and next-generation sequencing (NGS).

There are a few theories about the presence of cell-free nucleic acids in peripheral blood. Physiologically, 93% of cfDNA has an exosomal origin [36], and higher concentrations of cfDNA can be obtained from cells in the G1 phase of the cell cycle when cells can actively release DNA trapped in exosomal vesicles [37,38]. Interestingly, only a small amount of cfDNA might be secreted into the bloodstream within apoptosis of leukocytes and stromal cells [32,33,34], which happens when too many cells are involved in the apoptotic pathway, or the absorption capacity of the phagocytes is exhausted or inhibited [38]. On the other hand, ctDNA appears in circulation as a result of necrosis, apoptosis, or active release from a tumor [34,39]. However, in the ongoing neoplastic process, cancer cells can control the immune system through higher anti-apoptotic activity [9,40]. It has been also proven that ctDNA is more often detected than CTCs, thus confirming that ctDNA also has another origin than just CTCs [9]. Despite the fact that ctDNA consists of only some tumor-derived fractions of cfDNA, the actionable mutations in the liquid biopsy are limited to ctDNA. Thus, in oncologic studies, the term “ctDNA” is used synonymously with cfDNA to confirm to the reader that the study was performed on a liquid biopsy derived from an oncologic cohort. In this way, screening of mutations in ctDNA has the potential to be used in early cancer detection, to determine the prognosis and monitor response rate, and to assess potential resistance to the treatment, or to detect minimal residual disease (MRD) [41]. The possible mechanisms of tumor-derived DNA release and its clinical utility with the application of different genetic methods are summarized in Figure 1.

## 3. Liquid Biopsy—Diagnostic Issues

Liquid biopsy is considered to be a good non-invasive material that may be easily sampled; however, its diagnostic utility relates to the content and length of circulating acids in the bloodstream. The average length of cfDNA ranges between 180 and 200 base pairs (bp), and it may vary due to a very short half-life time—from 16 min to 2.5 h [42,43,44]. However, the length of ctDNA depends on many variables, such as the stage of the primary tumor or the type of applied therapy [43]. The length of the extracted cfDNA/ctDNA reflects the integrity of circulating DNA and, together with the concentration of cfDNA/ctDNA, may impact the sensitivity of diagnostic applications [37].

Currently, the most frequently chosen method for liquid-biopsy testing is the next-generation sequencing (NGS) and PCR-based approaches, as we presented in the Figure 1 [45,46,47,48]. NGS is a state-of-the-art technique that analyzes millions of short sequences, and it is characterized by its high sensitivity and specificity. If more than 180–200 bp cfDNA fragments are involved in the preparation of NGS libraries, the greater the sensitivity of their sequencing [43]. Therefore, it makes it easier to perform deep sequencing of targeted genomic areas rather than whole-genome sequencing (WGS) or whole-exome sequencing (WES) [46]. Numerous NGS-based methods offer a relatively broader screening of the genomic regions with high (>97%) sensitivity for very low allelic frequencies (~2%) [17]. There are also deep-sequencing approaches that allowed us to detection of 0.02% ctDNA mutant fractions with ~95% specificity in NSCLC. Deep sequencing also correlates well with the monitoring of the tumor-mutation burden (TMB), residual disease, or an early tumor response [49,50]. The second most frequently chosen method for the quantification of somatic mutations in the liquid biopsy is a targeted approach that focuses on “hotspots” for variation specific to a cancer type if the circulating DNA is in a proper range (180–200 bp) [46,47,48]. They include PCR-based methods such as droplet digital PCR (ddPCR) and BEAMing (beads, emulsions, amplification, and magnetics) that have shown a sensitivity range between 0.001 and 1% in detecting somatic point mutations [17,51]. PCR-based assays have shown promising results in sensitivity and specificity when using ctDNA that was further applied in clinical trials [17].

The ENSURE trial [52], published at the beginning of the previous decade, had proven the effectiveness of EGFR tyrosine kinase inhibitors (EGFR TKIs), namely erlotinib and gefitinib; in NSCLC patients with activating *EGFR* mutation detected in tumor tissue, that revolutionized the management of NSCLC treatment and has opened the era of personalized medicine in this field of oncology [53]. Soon after these observations, in 2016, the FDA approved the first quantitative PCR (qPCR) test for detecting an *EGFR* mutation in plasma cfDNA, which was concordant with tissue in 80% [54]. Therefore, patients with negative results of the mutation in the *EGFR* gene in liquid biopsy should be re-tested from the tissue sample [55,56]. Interestingly, the application of NGS techniques increased the concordance rate between these two diagnostic materials by only up to 85% [57]. However, the diagnosis of genetic abnormalities with standard PCR methods may be supplemented with wider molecular NGS panels, which target hot-spot genes, thus increasing the sensitivity of the analysis [58,59]. This proves that liquid biopsy is still not the ideal material for diagnostic manner, but its utility is invaluable if the primary tissue is scarce or unavailable, or if there are no possibilities to perform a re-biopsy. On the other hand, liquid biopsy indicated a high value in monitoring the response to TKIs in NSCLC [60].

## 4. Role of CTCs and ctDNA in Metastatic Spread

With the advancement of neoplastic disease, the amount of ctDNA in the bloodstream increases [61], and NSCLC may vary even 100 times between the I and IV stages of the disease [9]. Moreover, in advanced NSCLC patients, a higher concentration of ctDNA was detected, while metastases appeared outside the thorax [39]. On the other hand, a significant decrease in the amount of ctDNA was observed after resection was performed at the I or II stage of NSCLC [62]. There is also proof that both patients diagnosed with NSCLC and colorectal cancer (CRC) have significantly more cfDNA compared to the healthy control group; this may be caused by an intensive secretion of ctDNA fraction to the bloodstream during these tumors’ development [40]. Moreover, in advanced NSCLC, fragments of ctDNA are longer than they are at the early stage [39], and this may be due to necrotic cell decay in the tumor in advanced stages, as this also indicates the aggressiveness of the tumor [43]. What is more, long fragments of ctDNA could be related to tumor progression since chromatin does not shrink during necrosis; instead, it breaks randomly, creating fragments up to 10,000 bp [38,43,63].

During dissemination, the primary tumor cells need to gain the specific features that enable them to survive a long journey to a new site and facilitate colonization of the distant organs [64]. It seems that, at the beginning steps of metastatic spread, a crucial interplay exists between cancer cells and non-cancer components within the tumor microenvironment (TME) [65,66]. For instance, the cancer-associated fibroblasts (CAFs) support tumor growth by secretion of the extracellular matrix (ECM); IL-6 and IL-8 cytokines’; or TGFβ, HGF, GM-CSF, IGF, and VEGF growth factors [67,68]. Subsequently, the tumor-associated macrophages (TAMs) promote angiogenesis, tumor progression, metastasis, and resistance to chemotherapy under the influence of IL-4, IL-10, or IL-13 [69,70]. There are also cancer-associated adipocytes (CAAs) [69,71] and myeloid-derived suppressor cells (MDSCs) [72,73], that may modulate the cellular metabolism or angiogenesis processes within TME, respectively. Moreover, these cellular interactions in TME may be strengthened by epigenetic and transcriptomic processes that increase the metastatic potential of tumor cells, thus enabling them to be the released from their primary niche and survive within circulation as CTCs [66]. It was especially indicated that the hypo-methylation of *NANOG*, *OCT4*, *SOX2*, and SIN3A genes, which are responsible for pluripotency and proliferation of neoplastic cells, or the increased expression of CD44 (stem cell marker) on cancer cells increases the metastatic potential of CTC [74]. However, the formation of CTC clusters increases the risk of metastatic spread [75]. Biologically, CTC clusters are defined as small groups of tumor cells found in the bloodstream of cancer patients that may have a homotypic or heterotypic composition. In the first scenario, CTC clusters are composed of only tumor cells, while in the second scenario, CTC clusters also include nontumor cells, such as neutrophils, fibroblasts, platelets, or stroma-derived cells [76]. Recently, it has been described that the metastatic spread may be supported by neutrophil extracellular traps (NETs) composed of DNA strands, proteins, proteolytic enzymes, myeloperoxidase, or citrullinated histone 3 [77]. NETs sharpen tumor aggressiveness by enhancing cancer migration and invasion capacity by the entrapment of CTCs in the clusters, as well as triggering their proliferation within the metastatic niche [78].

It has also been proven that metastatic cfDNA activity is higher in cancer patients, rather than in healthy cohorts, by activation of components of the lipoprotein molecular complex [79]. The molecular complex may increase the interaction of lipoprotein receptors located on the normal cell surface with cancerous DNA, which leads to the transfection of normal cells located in the target organs with tumor-derived DNA [80]. In this way, ctDNA is involved in a mechanism of “genometastasis”, which involves the transfection of healthy cells with neoplastic ctDNA fragments [81,82,83], as well as the formation of cell colonies that are able to seed a distant metastatic niche [43,80,84,85]. This mechanism of neoplastic transformation mediated by ctDNA has been proved in fibroblastic cell cultures [86] or CRC and pancreatic-cell-line models [87,88]. It was also assumed that distant metastases in CRC may be formed as a result of the transfection of normal cells located in the target organs, with ctDNA harboring mutations in the *TP53*, *KRAS*, and *HBB* genes [89,90]. Furthermore, CRC-derived ctDNA increased, in vitro, the expression of the mRNA level of 118 genes, which included genes with metastatic potentials, such as *INSIG1*, *CREB3L2*, *CEACAM5*, *LIPG*, or *DAPP1* [91]. Moreover, it was confirmed that ctDNA may promote metastasis in recipient cells when toll-like receptor (TLR) pathways are activated by the overexpression of TLRs [91,92]. Therefore, cancers formed in the primary niche of the breast, ovarian, prostate, esophagus, and pancreas, in which cells harbor a high level of TLRs, are associated with worse prognosis and higher metastatic potential [92,93,94,95,96].

In NSCLC, it was proved that the ctDNA genetic landscape is affected by the clonality of the primary tumor, as it may affect the metastatic process [97]. An NSCLC TRACERx study showed that the size of the primary tumor correlated with the higher number of clonal variants. However, the probability to detect subclonal variants in ctDNA samples increased with the spread of these variants in the different primary NSCLC regions, as well as in patients who develop tumor relapse [98]. Interestingly, the subclonal variants were detected in plasma even 70 days earlier than tumor relapse was indicated in radiological imaging procedures [99]. These observations indicate a high potential value that liquid biopsy may be brought into clinical practice for tracking the tumor relapse and clonal heterogeneity that sculpt the evolution of NSCLC.

## 5. Role of Liquid Biopsy in the Monitoring of Response to Personalized Therapies

In addition to the involvement of ctDNA in the process of cancer spread and formation of distant metastases, there is many proof of its practical application in clinical routine at each stage of cancer development [100]. There is especially a trend to broaden the implementation of liquid biopsy for the noninvasive monitoring of personalized treatment [101]. Several studies simply evaluated the changes in total cfDNA level within the treatment, showing that the absence of cfDNA correlates with a better response to the treatment and a longer progression-free survival (PFS) and overall survival (OS) compared to a situation when cfDNA is detectable at a high level before treatment [102,103]. Metastatic CRC concentrations of cfDNA > 26 ng/mL and ctDNA > 2 ng/mL are correlated with a shorter OS in patients [104]. Similarly, a melanoma concentration of ctDNA > 23.6 ng/mL was associated with a higher risk of progression and death [105]. In breast cancer, the concentration of ctDNA at 120 ng/mL may be considered an early indicator of cancer advancement in screening tests [106]. In general, Tissot C. et al. indicated that NSCLC patients with higher cfDNA concentrations (threshold = 42.12 ng/uL) at the baseline of therapy had a significantly shorter OS than patients with lower cfDNA levels (median OS: 10 months vs. 14.2 months, respectively) [107]. Similarly, Dziadziuszko R. et al. suggested that cfDNA had a prognostic value in advanced *ALK*+ NSCLC [108]. However, due to a lack of strong proof, this observation measurement of the cfDNA level at baseline in NSCLC patients was not applied to the clinical routine [109]. Moreover, at the early stages of the disease, after complete surgical resection cfDNA, concertation can be used for detection of the tumor relapse [44] or monitoring the genetic background of residual disease [110]. There are many premises that liquid biopsy may be useful for monitoring the changeable landscape of “druggable” biomarkers for targeted therapies or immunotherapies [111]. There are already, in total, 39 clinical trials evaluating the utility of liquid biopsy in NSCLC patients, indicating a huge interest in the clinical applicability of this material. In Table 1, we briefly summarize 19 clinical trials that are already recruiting NSCLC patients.

### 5.1. Liquid Biopsy in the Monitoring of Molecularly Targeted Therapies

The first study on the effectiveness of liquid biopsy in the monitoring of personalized treatment associated the appearance of a higher number of mutated alleles in liquid biopsy with the emergence of resistance to the applied treatment [113]. Since then, a liquid biopsy was widely used in monitoring response to molecularly targeted therapies in many solid tumors, such as breast cancer [114], colorectal cancer [115], pancreatic cancer [116], and head and neck cancer [117]. However, its great impact was recently valued in NSCLC [60] when a liquid biopsy was applied for analysis of “druggable” abnormalities such as mutations in *EGFR* [118] and *BRAF* [119] genes, as well as *ALK* and *ROS1* rearrangements [120] or *MET* amplification [121].

The highest clinical utility in NSCLC liquid biopsy was proved in monitoring the level of *EGFR* gene mutations during the first-line treatment with first (erlotinib, gefitinib) or second (afatinib, dacomitinib) generation of EGFR TKIs [7,122]. Liquid biopsy especially has a great value for early detection of T790M substitution in exon 20 of the *EGFR* gene that confers the resistance to the first two generations of EGFR TKIs and simultaneously determines the sensitivity to osimertinib—the third generation of EGFR TKIs [123]. The AURA clinical trial confirmed that, in NSCLC patients harboring T790M substitution, the administration of osimertinib may overcome the acquired resistance to TKIs [124]. Therefore, early detection of T790M mutation in the liquid biopsy of NSCLC allows us to adjust the treatment regimen and continue the effective therapy [125]. In one of our studies, we also indicated that sequential evaluation of *EGFR* gene status in consecutive liquid biopsies, using a qPCR technique, has a high value in monitoring the response to EGFR TKIs and allows for early detection of acquired resistance determined by T790M substitution when disease progression is not detected by computed tomography yet [55]. However, despite the methodological advancement, evaluation of cfDNA for T790M mutation indicates 70% sensitivity, and even 30% of patients with negative results require a re-biopsy of a progressed tumor, and this, in many cases, is challenging [126]. There are also some reports stating that, in NSCLC patients with *ALK* gene rearrangement, the L1196M and S1206Y substitutions in the *ALK* gene may be screened in liquid biopsy as possible factors of resistance to crizotinib—the first line of ALK TKI [127]. Moreover, there are many studies describing the potential clinical utility of mutation testing in the liquid biopsy of NSCLC patients in *ROS1* [128,129], *MET* [130,131], and *BRAF* [119,131] genes.

### 5.2. Monitoring of Immunotherapy

In recent years, immunotherapy based on immune checkpoint inhibitors (ICIs) has also become an important tool in the personalized treatment of NSCLC [132]. The efficacy of ICIs was especially observed in heavy smokers, and it was initially related to the high expression of tumor-specific neoantigens and high tumor-mutation burden (TMB) induced by tobacco carcinogens [133,134]. TMB is defined as the number of total mutations in the tumor and is the most common predictor of response to ICIs after anti-PD-L1 immunohistochemistry staining in the USA [135]. However, it is debatable which mutations trigger the immunogenicity by neoantigen [125]; thus, the expression of PD-L1 on NSCLC cells remains a key clinical biomarker of responsiveness to ICIs [136]. Despite many advantages of ICIs, NSCLC patients respond differently to immunocompetent agents: 10–15% of NSCLC patients show a very long-term response (longer than 5 years), 40–50% of patients indicate a primary resistance, and 25–35% of patients acquire the resistance within the first 6–12 months of treatment [137]. The primary and acquired resistance to ICIs develops in different mechanisms and may be associated both with genetic background and impaired function of the immune system that facilitate tumor cells to escape from immune surveillance [138]. However, there is limited knowledge about the genetic biomarkers of resistance to ICIs that could be used for tracking the response rate in liquid biopsy.

Similar to molecularly targeted therapies, there are attempts to define the association between the baseline level of cfDNA and response rate to immunotherapy. In NSCLC, there were no significant differences between the ctDNA level and OS during immunotherapy. However, the initial response to ICIs may be indicated early on, at the cfDNA level, rather than in a radiological image. Additionally, a decrease of cfDNA concertation within the first 8 weeks of immunotherapy correlates with a better response rate [139]. On the other hand, the constant cfDNA concentration within the initial treatment period resulted in faster disease progression [140]. There are also attempts to apply to monitor TMB in liquid biopsy (blood TMB; bTMB), which is concordant with tissue TMB (tTMB) in 70% and seems to be more representative of the entire neoplastic process [141]. There are some discrepancies between clinical thresholds for bTMB and tTMB in NSCLC patients. For instance, Ttmb > 10 mutations/Mb was associated with a shorter PFS and OS during the implementation of ICIs, while a similar relationship was observed for bTMB > 6 mutations/Mb [142]. However, a low level of bTMB was insignificantly correlated with longer PFS or OS in NSCLC patients treated with ICIs, while NSCLC patients with high bTMB may benefit from ICIs compared to patients who received chemotherapy [143,144]. Moreover, a higher bTMB level is observed in metastatic NSCLC, while a lower bTMB is more common for the early stages of NSCLC [145]. Recently, bTMB has found large interest in many clinical trials carried out in NSCLC, as we summarize in Table 2.

## 6. Conclusions and Further Perspectives

Liquid biopsy indicates a huge potential in the monitoring of treatment response, cancer progression, or relapse, which may improve the management of NSCLC. On the other hand, the interest in liquid biopsy in NSCLC is limited in clinical trials, as it is geared toward the early stages of the disease, where the evaluation of minimal residual disease after surgical resection is one of the major clinical challenges [149]. The longitudinal assessment of genetic alterations in plasma has the potential to provide results about predictive biomarkers of acquired resistance, especially when tissue from prospective re-biopsy is rarely available. However, implementation of liquid biopsy testing at early stages has some limitations that result from (i) the low amount of ctDNA at an early stage; (ii) dilution of cancerous allele frequency by alterations associated with clonal hematopoiesis; and (iii) the fact that cancerous mutations might not be specific to the particular cancer type, and thus tissue of origin based on mutated ctDNA is often uncertain [149]. The recent breakthrough in NGS approaches increased the sensitivity of liquid biopsy testing. There are also promising reports that ctDNA evaluation after a positive CT scan could improve the overall accuracy of NSCLC screening and reduce the number of invasive procedures. It is possible that ctDNA testing may completely replace low-dose CT for lung-cancer screening [150,151]; however, such wide sequencing implementations to the clinical routine would need to consider the cost and time of analysis.

Future applications of liquid biopsy are likely to concern the study of primary resistance to treatment in order to optimize therapy personalization. Due to the fact that a large spectrum of elements may be measured by liquid biopsy, it may be used to monitor many more biological changes of the disease during the treatment than are currently being proposed. For instance, the analysis of cfDNA methylation may be applied to distinguish between non-tumor and tumor cfDNA [152], and there are many deregulations at the microRNA level that are small non-coding RNA levels whose incidence has not been described yet [60]. Moreover, the interplay between liquid biopsy and TME should be further studied as a potential predictive marker of immunotherapy and tumor evolution. However, future directions for liquid biopsy in NSCLC relate to CTCs, which may be potentially useful for the study of PD-L1 expression [153,154,155]. To date, the studies on CTCs have not provided any spectacular results and have not been associated with the prediction of response to the therapy. A low number of CTCs in a single liquid biopsy and technical challenges in CTCs detection are the main clinical or diagnostic limitations in this matter.

However, interest in CTC provides an interception concept that aims to thwart the development of primary tumors or their metastases. The concept includes the prevention processes in high-risk individuals by (i) identification and elimination of risk factors associated with carcinogenesis, (ii) detection of cancer driver gene mutations/biomarkers, and (iii) implementation of all necessary procedures to create the early detection programs [151,156,157]. Another field of the setting of cancer interception and prevention includes an interest in the predictive role of circulating miRNA in lung-cancer patients as a potential alternative to low-dose computed tomography (LDCT) or to more invasive screening procedures [158]. Moreover, the use of ctDNA as a surrogate marker of residual disease before metastases become clinically detectable is attractive for early cancer interception, too [149].

To summarize, cfDNA is currently associated with greater hope for common diagnostic and clinical applications. However, research on the adoption of liquid biopsy for cancer interception is still limited, and it remains to be determined if liquid biopsy may play a crucial role in asymptomatic-cancer detection. We believe that the wide scientific application of single-cell sequencing and spatial transcriptomics is an important step closer to the clinic.

## Figures and Tables

**Table 1:** A summary of clinical trials that are already recruiting NSCLC patients to evaluate the applicability of liquid biopsy for monitoring the response rate to personalized treatment or disease recurrence after surgery. Data were collected from the ClinalTrials.gov database [112].

Clinical Trials ID (Duration)	Number of Participants (Trial Type)	Applied Technique	Primary Outcomes
NCT04703153 (04.2021–11.2023)	200 (observational)	NGS (Comprehensive Genome Panel)	To explore the non-inferiority of liquid biopsy vs. tissue biopsy by NGS assay for genetic-alteration profiling in NSCLC patients who have detected abnormality (in tissue biopsy) in at least one of nine common actionable genes (*EGFR*, *ALK*, *RET*, *ROS1*, *NTRK*, *MET*, *BRAF*, *ERBB2*, *KRAS*).
NCT04474613 (08.2020–11.2023)	100 (interventional)	No information	To examine if using liquid biopsy can reduce time to treatment in newly diagnosed NSCLC patients who have not had genetic testing for targetable mutations. The study will examine the time to actionable genetic testing results (ctDNA or tissue), rate of actionable biomarker discovery, and rate of appropriate guideline-directed therapy based on testing results.
NCT04912687 (01.2022–01.2024)	580 (interventional)	ddPCR/qPCR (*EGFR* gene)	To evaluate the detection rate of EGFR gene mutation in newly diagnosed patients with advanced NSCLC in a real-world clinical setting, based on liquid biopsy and tissue analyses.
NCT04863924 (01.2021–12.2023)	170 (observational)	No information	To assess the utility of liquid biopsy to accelerate the time to treatment for patients with newly diagnosed advanced NSCLC, compared to the conventional diagnostic pathway of molecular testing of tumor tissue after imaging and biopsy.
NCT02511288 (07.2015–12.2026)	900 (observational)	ddPCR + targeted NGS (*EGFR*, *BRAF*, *HER2* mutations; *ALK*, *ROS1* translocation, *MET* amp, *RET* rearrangement)	To identify the genetic profile in advanced or metastatic NSCLC patients by using liquid biopsies. Evaluation of the liquid biopsies’ role in tumoral monitoring.
NCT04790682 (05.2021–05.2024)	300 (interventional)	NGS (type not defined)	The assessment of the predictive value of ctDNA in the monitoring of the prominent mutant allele variation between baseline and after 6 weeks of treatment in NSCLC patients receiving pembrolizumab in monotherapy.
NCT04258137 (09.2020–04.2024)	332 (interventional)	NGS (type not defined)	To provide a therapeutic recommendation based on tumor sequencing and then follow-up combining standard imaging and ctDNA analysis based on tumor sequencing and then follow-up based on standard imaging in NSCLC patients receiving various treatment regimens.
NCT05254782 (07.2021–06.2025)	360 (observational)	ctDNA detection (method not defined)	To assess the ctDNA detection rate and its association with Relapse-Free Survival in surgical stages of NSCLC patients.
NCT03865511 (04.2019–07.2024)	66 (interventional)	Tissue biopsy: ddPCR (C797S); FISH; (*MET* amp); IHC (PD-L1, CD73, CD4, CD8); NGS (deep sequencing aimed to call acquired mutations and CNAs) Liquid biopsy: ddPCR (*EGFR* activating mutation); NGS (type not defined)	To study the association between changes of genetic parameters in ctDNA to predict the response to osimertinib or to define the mechanism to resistance to osimertinib in NSCLC patients with EGFR activating mutation receiving osimertinib in the first line of treatment.
NCT03774758 (12.2017–12.2023)	590 (observational)	NGS (Guardant Health, panel not defined)	Estimation of clinical sensitivity and specificity of the ctDNA assay to follow patients who undergo lung-cancer screening. The study will comprise two populations selected by CT scan to determine the ctDNA assay performance in a variety of clinical settings.
NCT04957602 (10.2021–06.2023)	40 (observational)	Microfluidic CTC extraction	Evaluation of if CTCs may replace tissue biopsies in the prediction and monitoring of therapeutic responses and tumor recurrence in metastatic NSCLC patients who have not initiated their treatment yet (osimertinib or chemotherapy).
NCT04966663 (03.2022–08.2025)	66 (interventional)	ctDNA detection (method not defined)	To evaluate if the level of ctDNA in the blood may help to predict the risk of cancer recurrence in surgical stages NSCLC in patients who received adjuvant chemo-immunotherapy.
NCT05221372 (02.2017–01.2031)	1300 (observational)	NGS (custom amplicon-seq; Oncomine Lung cfDNA Assay v1; Thermo Fisher Scientific)	To collect repeated samples of liquid biopsy from NSCLC patients (starting) on TKIs, for testing, and pharmacokinetic analysis of mutations.
NCT05145244 (08.2021–12.2023)	2400 (observational)	NGS (whole-genome sequencing)	To perform refined biomarker analyses on tumor and liquid biopsies in NSCLC patients receiving treatment offered in the clinic (standard of care or included in clinical trials) to identify new potential treatment targets.
NCT04976296 (09.2021–12.2027)	300 (observational)	cfDNA detection (method not defined)	To investigate the value of MRD for stage I-IIIA NSCLC patients who underwent complete resection. Preoperative blood samples, tumor tissue, and dynamic postoperative blood samples collected continuously every 3–6 months will be used for MRD detection.
NCT05059444 (09.2021–10.2027)	1000; various solid tumors, including NSCLC (observational)	ctDNA detection (method not defined)	To demonstrate the ability of a novel ctDNA assay to detect recurrence in individuals treated for early stage solid tumors. The ctDNA test results will be linked to clinical outcomes to demonstrate clinical validity for recurrence detection and explore its value in a healthcare environment subject to cost containment.
NCT04566432 (07.2020–07.2023)	250 (observational)	Detection of mutation in ctDNA (method not defined)	To evaluate the predictive value of ctDNA for response or relapse in NSCLC patients treated with immune checkpoint inhibitors or targeted therapy for *ALK* and *ROS1* rearrangements and *MET* ex14 skipping mutation.
NCT05020275 (12.2021–12.2023)	60 (observational)	Blood concentration of ctDNA and Osimertinib (method not defined)	To evaluate the relationship between the plasma concentration of osimertinib and ctDNA with response rate (PFS) in NSCLC patients with EGFR-activating mutations.
NCT04564079 (06.2021–08.2023)	200 (observational)	NGS (amplicon-seq based on Oncomine Precision Assay; Thermo Fisher Scientific)	The study will evaluate the clinical utility of Oncomine Precision Assay for monitoring the recurrence of genomic aberration in blood and/or tissue in patients with IIIb/IV stage of NSCLC.

**Table 2:** The summary of clinical trials that evaluate the applicability of bTMB testing in liquid biopsy for monitoring the response rate to ICIs. Data were collected from the ClinalTrials.gov database (112).

Clinical Trial ID (Duration)	ICIs Tested	Target of Research/Conclusions
NCT02848651 (B-F1RST) [146] (07.2016–05.2019)	Atezolizumab	B-F1RST shows the clinical utility of bTMB as a predictive biomarker for patients receiving first-line atezolizumab monotherapy. The final analysis confirmed that patients with bTMB ≥ 16 had a benefit for PFS and OS.
NCT02542293 (NEPTUNE) [147] (11.2015–12.2022)	Tremelimumab	bTMB ≥ 20 was associated with a longer OS.
NCT02409342 (IMpower110) [148] (07.2015–03.2022)	Atezolizumab	bTMB≥16 correlated with high PD-L1 expression and better response
NCT03178552 (BFAST) (09.2017–04.2024)	Atezolizumab	The ongoing study determines the impact of oncogenic somatic mutations or positive bTMB on different personalized treatment
NCT04765709 (BRIDGE) (09.2021–06.2026)	Durvalumab	The ongoing study to assess the dynamic changes bTMB has upon different treatment time points.

## Data Availability

Not applicable.
